# Variant interpretation in molecular autopsy: a useful dilemma

**DOI:** 10.1007/s00414-021-02764-z

**Published:** 2022-01-29

**Authors:** Stefanie Scheiper-Welling, Monika Tabunscik, Theresa E. Gross, Tina Jenewein, Britt M. Beckmann, Constanze Niess, Elise Gradhand, Cora Wunder, Peter M. Schneider, Markus A. Rothschild, Marcel A. Verhoff, Silke Kauferstein

**Affiliations:** 1 Institute of Legal Medicine, Goethe University, University Hospital Frankfurt, Frankfurt, Germany; 2 Institute of Legal Medicine, Faculty of Medicine and University Clinic, University of Cologne, Cologne, Germany; 3 Institute of Pathology, Goethe University, University Hospital Frankfurt, Kennedyallee 104, 60596 Frankfurt, Germany

**Keywords:** Sudden death, Sudden cardiac death, Post-mortem genetic screening, Molecular autopsy, VUS, Inherited arrhythmic syndrome

## Abstract

Sudden cardiac death (SCD) in adolescents and young adults may be the first manifestation of an inherited arrhythmic syndrome. Thus identification of a genetic origin in sudden death cases deemed inconclusive after a comprehensive autopsy and may help to reduce the risk of lethal episodes in the remaining family. Using next-generation sequencing (NGS), a large number of variants of unknown significance (VUS) are detected. In the majority of cases, there is insufficient evidence of pathogenicity, representing a huge dilemma in current genetic investigations. Misinterpretation of such variants may lead to inaccurate genetic diagnoses and/or the adoption of unnecessary and/or inappropriate therapeutic approaches. In our study, we applied current (ACMG) recommendations for variant classification in post-mortem genetic screening of a cohort of 56 SCD victims. We identified a total 53 rare protein-altering variants (MAF < 0.2%) classified as VUS or worse. Twelve percent of the cases exhibited a clinically actionable variant (pathogenic, likely pathogenic or VUS – potentially pathogenic) that would warrant cascade genetic screening in relatives. Most of the variants detected by means of the post-mortem genetic investigations were VUS. Thus, genetic testing by itself might be fairly meaningless without supporting background data. This data reinforces the need for an experienced multidisciplinary team for obtaining reliable and accountable interpretations of variant significance for elucidating potential causes for SCDs in the young. This enables the early identification of relatives at risk or excludes family members as genetic carriers. Also, development of adequate forensic guidelines to enable appropriate interpretation of rare genetic variants is fundamental.

**Supplementary Information:**

The online version contains supplementary material available at 10.1007/s00414-021-02764-z.

## Introduction

Sudden cardiac death (SCD) is defined as a sudden and unexpected natural death caused by cardiac origin. Previous studies reported an overall SCD incidence rate of 15 to 159 per 100.000 per year [1]. Both incidence and cause differ markedly with the age of the victim, whereas SCD accounts for 20% of deaths among young individuals [2–6]. However, the incidence reported in official mortality statistics of young individuals who died suddenly and unexpected is certainly underestimated. A significant proportion of SCDs in young individuals (1–35) is caused by inherited arrhythmia syndromes and inherited cardiomyopathies [3, 7–11]. Thus, genetic analyses may help to determine the cause of sudden unexpected death cases in the young, deemed inconclusive after a comprehensive autopsy. Moreover, it is well accepted that cardiological and genetic screening of family members in combination with molecular autopsy in the deceased individual can be enlightening and help to reduce the risk of further lethal casualties within the remaining family.

Recent advances in next-generation sequencing technologies allow a more rapid and less expensive sequencing of numerous genes at once as well as sequencing of the whole exome or entire genome. To guarantee a high level of care within the cardiogenetic evaluation in SCD cases and the affected families, it is important to emphasize that forming a multidisciplinary team is an essential key aspect [12–15]. An important task of a multidisciplinary team is the interpretation of variants detected during a molecular autopsy or family cascade screening. Variant interpretation, the process of determining whether a DNA variant may be causative, relies on multiple lines of evidence. Final variant classification depends on the interpretation of evidence. For instance, the prevalence of the disease and – in relation to this – the population frequency of the variant is one important issue. Computational and predictive analysis (including preservation of the wild type amino acid across different species or functional relevance of the domain affected) as well as functional studies and co-segregation of the variant with a clinical phenotype must be taken into account. It has to be kept in mind that genetic testing is never a deterministic but a probabilistic test.

The American College of Medical Genetics and Genomics (ACMG) published guidelines for the assessment of sequence variations and established five categories of variant classification, ranging from pathogenic to benign in order to enable comparable variant interpretation between laboratories [16]. While some variants can be confidently predicted as pathogenic or benign, in many cases, only insufficient information can be provided. These variants are classified as variants of unknown significance (VUS). With the introduction of high-throughput technologies, the number of VUS is growing exponentially, leaving the affected families and their physicians in the so-called “genetic purgatory” [17]. Classification of a VUS can be resolved over the time, as more data is gathered and the VUS will be eventually reclassified to pathogenic or benign. For instance, one common step in solving the interpretation of a VUS is testing of family members to determine whether variants are shared by other affected or unaffected individuals. However, misinterpretation of a rare variant may lead to a false presumed cause of death or genetic diagnosis and subsequently the adoption of unnecessary approaches. There are some cases published, showing the devastating impact of an incorrect interpretation of a variant-disease relationship [13]. Hence, interpretation of genetic results in a young SCD victim and consecutively their families is challenging, but important. Experienced multidisciplinary teams are necessary for an adequate interpretation of the genetic and possible clinical findings in the decedents and relatives as well as for further recommendation on therapy and family counselling. In conclusion, VUS are a dilemma for subsequent variant interpretation, but useful for discussing these especially ambiguous cases in a multidisciplinary team and implementing measures in affected families, which may help to identify family members being at risk.

In this study, we describe the investigation of 56 unrelated sudden death cases in the young. We investigated the genetic basis of sudden death and the prevalence of variations in genes associated with cardiac disease using massively parallel sequencing. We also performed proper variant assessment on a case by case basis in an experienced multidisciplinary team.

## Material and methods

### Study subjects

In this study, we retrospectively collected data from 56 sudden death (SD) victims, aged between 1 and 50 years. The investigated cases from the years 2010 to 2020, mainly received from the Institutes of Legal Medicine in Frankfurt and Cologne. SD was defined as a sudden, natural, and unexpected death (SUD). The person had to be seen alive and in a good healthy condition < 24 h before being found dead or in witnessed cases; the death had to be occurred within 1 h of change in cardiovascular status. A medico-legal autopsy was performed in every case. If cardiac tissue and blood were available, histological as well as chemical-toxicological examinations were performed. We excluded SD cases, if death happened in correlation with acute drug abuse or myocardial infarction. Toxicological-positive findings for therapeutic drugs had to be in accordance with the medical history. Pre-existing conditions were not an exclusion criterion, if the time and manner of death were unexpected. If no explanation of the death was found by autopsy including histological examination and toxicological investigations as well as non-cardiac etiologies were excluded, the term SADS (sudden arrhythmic death syndrome) was used. Heart weight was calculated by using the Chicago model for post-mortem classification of cardiomegaly (https://labs.feinbergnorthwestern.edu/webster/heart_weight/).

The current study was approved by the ethical commission of the University Hospital, JWG University Frankfurt (protocol number E169/06).

### Sample preparation

DNA was isolated from autopsy whole blood or tissue samples using NucleoSpin Tissue Kit (Macherey Nagel) with minor optimizations in comparison to manufacturer’s recommendations: pre-lysis incubation overnight, doubled centrifugation time, 2 min instead of 1 min, following the DNA binding, washing and drying step of the protocol as well as a reduced elution volume, 50 µL instead of 100 µL, in the final step.

DNA integrity was determined using the Agilent 4200 TapeStation (Agilent Technologies) and the genomic DNA ScreenTape. For samples exhibiting a DNA integrity number (DIN) lower than 5, 300 ng of DNA was used as input for tagmentation, and above a DIN of 5, 200 ng was used. In case of very limited sample amounts and/or concentrations, at least 50 ng served as input.

### Post-mortem genetic analysis

The DNA of all cases was analyzed for sequence variations using the TruSight cardio panel (Illumina) consisting of 174 genes with known cardiac associations. Paired-end libraries were prepared following the manufacturer’s protocol Nextera™ Flex for Enrichment as described previously [18, 19]. Sequencing was performed on the Illumina® MiniSeq™ system (2 × 150 bp paired end reads).

### Data analysis

Resulting reads were aligned to the human reference genome GRCH37/hg19. Variant calling and evaluation were performed using GensearchNGS software (PhenoSystems). The genetic data were filtered for aberrations in genes associated with cardiac channelopathies and cardiomyopathies (*n* = 93, supplemental data). Only variants were included, if they showed a high-quality score (quality of bases supporting the variant showed at least a base phred of 20, accuracy of 99%) and exhibited a minor allele frequency (MAF) < 0.2% within the Genome Aggregation Database (gnomAD) [20] and the 1000 Genomes Project [21]. Genetic variants expected to affect or disrupt protein function, e.g., those located in exonic or splicing flanking regions as well as insertions/deletions were selected and those referred to as synonymous excluded. Variants meeting these criteria were assessed using common databases (Genome Aggregation Database, NHLBI Exome Sequencing Project, NCBI dbSNP, Human Gene Mutation Database) and applying in silico prediction tools (PolyPhen-2 [22], MutationTaster [23], SIFT [24] as well as CADD [25]).

The guidelines of the American College of Medical Genetics and Genomics (ACMG) [16] were used to classify detected sequence variations as pathogenic (P), likely pathogenic (LP), variant of uncertain significance (VUS), likely benign (lb), or benign (b). Detected sequence variations were designated according to the nomenclature recommendations by the Human Genome Variation Society (HGVS, https://varnomen.hgvs.org/). Variants expected to disrupt protein function (nonsense, frameshift, canonical splice site variants) were classified as important variants (potentially pathogenic). Furthermore, rare missense variants within the core genes (supplemental data) were classified as important variants, when the reference amino acid was highly conserved; the variants were located in a functionally important domain, in mutation hotspots or described in this context in literature.

For the final classification of the variants – especially for those with ambiguous significance – we discussed the cases including medical, histological (where appropriate toxicological), and genetic data in a team of forensic pathologists, pathologists, toxicologists, cardiologists, and geneticists. In order to obtain more background information, we also contacted further physicians of the deceased and family members.

## Results

### SD cohort

The descriptive data of the analyzed cohort are summarized in Table 1. The cohort of suspected SUD victims included 56 deceased (18 females/38 males; aged 1–50 years). In 13 cases, there was a personal history of mild drug abuse, but none exhibited a lethal concentration of drugs in the blood prior to death (Table 1). Twelve percent of the cases had a personal history of some mental illness.

**Table 1:** Cohort demographics (*N* = 56)

Characteristics	All *(n* = 56*)*	
Gender (female/male)	19/38	(33%/67%)
Median age in years (range)	29	(4–50)
BMI > 30	18 (*n* = 52)	(35%)
Toxicology screening	36	(63%)
Negative	23	(64%)
Antipsychotics	1	(3%)
Antidepressants	1	(3%)
Illegal drugs	6	(17%)
Analgesics	3	(8%)
Antiepileptic drugs	2	(6%)
Increased heart/body weight ratio	21 (*n* = 54)	(39%)
Syncope prior to death	2	(4%)
Arrhythmia	3	(5%)
Decreased cardiac output	1	(2%)

**Fig. 1:**
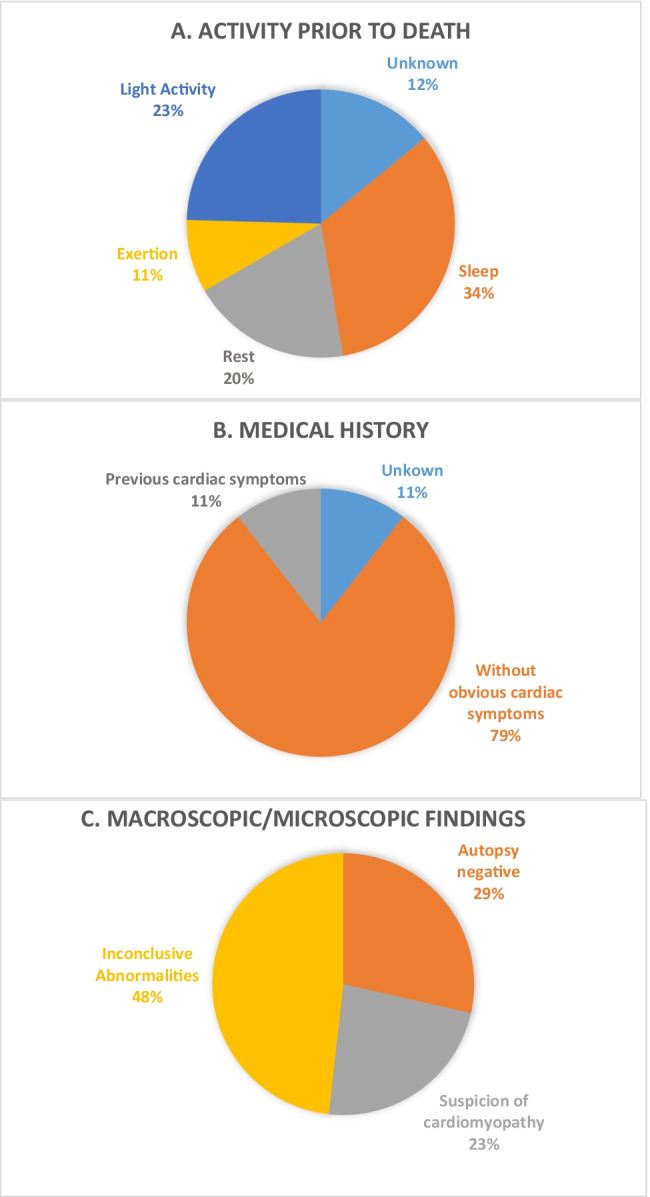
Characteristics of the SUD probands (*n* = 56). (**A**) Activity prior to/circumstances of death. (**B**) Reported medical history of the deceased. (**C**) Cardiopathological autopsy findings

Sudden death was witnessed in 49 of 56 cases, whereas death was unwitnessed in the remaining 7 cases (Fig. 1a). The sudden death victims died either during sleep (34%), rest (20%), exertion (11%), or light activity (23%).

Cardiac symptoms prior to death (arrhythmia, heart failure, or syncope) were described in 11% of the cases. For instance, in two cases, arrhythmia occurred prior to death, whereas in one case, Torsade de Pointes tachycardia was recorded in the patient’s night of death, and in another case, the deceased woman told her sister that she suffers from a decreased cardiac output; no further information was given (Fig. 1b).

In 29% of the cases, the autopsy was without any pathological findings (negative). Enlarged hearts in comparison to international standards were observed in 21% of the cases. In total, 50% of the cases had equivocal cardiopathological findings or inconclusive abnormalities (Fig. 1c).

### Genetic analysis of sudden death susceptibility genes

**Fig. 2:**
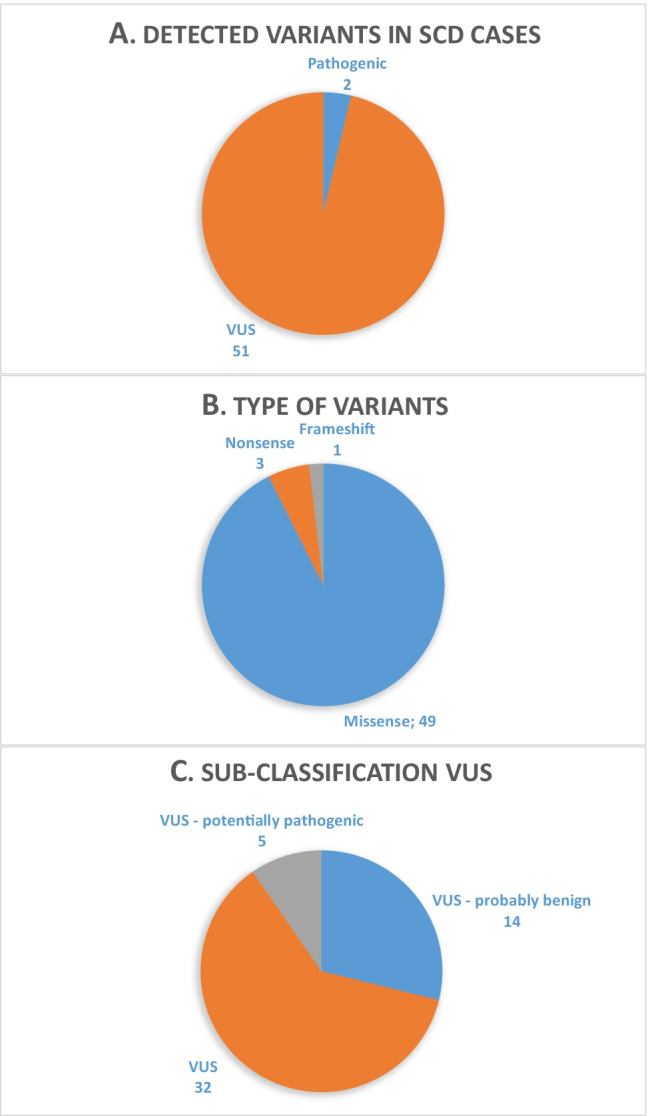
Overview of detected variants in the SUD cohort. (**A**) Classification of identified variants. (**B**) Distribution of mutation types of the 53 evaluated variants. (**C**) Further sub-classification of the 51 VUS

Genetic testing revealed a total of 53 rare protein-altering variants (MAF < 0.2%) in 32 different genes out of the 93 genes investigated and associated with inherited arrhythmogenic disease. While 17 of the variants were identified in core genes (i.e., strong evidence for disease association), another 36 were detected in minor genes (i.e., lower detection rate of disease-associated variants or limited evidence for disease association, respectively). Eleven cases comprised more than one rare variant. Applying the ACMG guidelines, two rare variants were classified as pathogenic (Fig. 2A, Table 2). Most of the variants found in our study were missense variations, categorized as VUS (Fig. 2B). Further sub-classification (Fig. 2c, Table 2) revealed that 5 VUS may be potentially pathogenic (rare, hosted in a core gene, genotype–phenotype concordance if available, described in SCD cases, high scores of in silico prediction indicating pathogenicity). Twenty-two cases (39%) exhibited no sequence variations. In cases with a pathogenic or potentially pathogenic variant, no drugs associated with LQT prolongation could be detected.

**Table 2:** Investigated cases showing genetic variants with potential relevance

Index	Sex	Age	Setting	Gene	Nucleotide	Protein	dbSNP	MAF (%)	CADD	ACMG variant classification
28	F	14	Sleep	*KCNH2*	c.1600C > T	p.(Arg534Cys)	rs199472916	NA	25	Pathogenic
43	M	26	During 10 km run	*SCN5A*	c.127C > T	p.(Arg43*)	rs1553607597	NA	36	Pathogenic
13	F	24	Sleep	*KCNQ1*	c.1555C > T	p.(Arg519Cys)	rs199472787	0,00004	31	VUS (potentially pathogenic)
33	M	39	In gym during exercise	*KCNH2*	c.1591C > T	p.(Arg531Trp)	rs199472915	NA	26,9	VUS (potentially pathogenic)
53	F	42	Sleep	*MYBPC3*	c.2993A > G	p.(Gln998Arg)	rs727503177	NA	34	VUS (potentially pathogenic)
20	M	27	Everyday activities	*VCL*	c.1558C > T	p.(Arg520Trp)	rs77722209	0,002	31	VUS (potentially pathogenic)
56	M	50	Everyday activities	*JUP*	c.608G > A	p.(Arg203His)	rs200221163	0,003	32	VUS (potentially pathogenic)

In the SUD cases with a clinically actionable variant (pathogenic or likely pathogenic, VUS potentially pathogenic), the family was contacted to provide recommendations and options for further management.

## Discussion

The majority of the 56 young sudden death victims investigated in this study occurred during sleep and physical exercise, which is in accordance with previously published studies [7, 8, 26–28]. Cardiac syndromes responsible for SUD are generally characterized by autosomal inheritance, locus heterogeneity, incomplete penetrance, and variable expression [29]. At present, more than 100 genes are associated with diseases leading to sudden unexpected death. The process of interpreting the pathogenicity of a variant properly aside from correct variant calling is an important task of comprehensive cardio-genetic evaluation after a SUD as confirmed in our study to enable appropriate counselling of the families.

The American College of Medical Genetics and Genomics (ACMG) and the Association for Molecular Pathology (AMP) published a guideline that provides a framework for sequence variant interpretation based on in silico predictions, global population frequencies, and functional in vivo/in vitro analysis. The terms “pathogenic” and “likely pathogenic” describe variants that have further implications for diagnostic purposes and family counselling [16]. Seven variants of our study were determined as pathogenic, likely pathogenic, or VUS potentially pathogenic and thus are “clinically actionable”.

Most of the variants we detected in our genetic investigations were variants with unknown significance (VUS). A VUS based on the ACMG/AMP framework is an inconclusive result if there is either insufficient or conflicting evidence regarding the pathogenicity. However, the current ACMG guidelines have not been independently validated, especially when used in a forensic context.

The cases in our study were collected retrospectively, mainly based on autopsy results and police records. Hence, medical history or detailed heart pathology as well as family investigations was not available. Therefore, detection of a VUS is a highly likely result of a molecular autopsy in forensic practice, because the assignment of the detected variant’s significance is not possible due to the lack of supporting background data.

As the VUS by itself does not provide any evidence or a molecular confirmation of a potential diagnosis, it is reasonable to conclude that most of the variants detected in our study have no effect on protein function and thus might not be “potentially informative”?

A range of prediction tools are available to address this question, but these often suffer from poor specificity and consequently result in highly false-positive rates. Although functional testing can give some insights about a variant’s clinical validation [30–32], it is insufficient to conclude that altered protein function is the probable underlying cause of death.

Thus, genetic testing by itself might be fairly meaningless if a VUS is detected. There are currently no specific forensic guidelines on the management and interpretation of SUD cases where a VUS has been detected. Nevertheless, the number of VUS is growing in the field of forensics. Therefore, we recommend working with experienced multidisciplinary teams required for the proper interpretation of the genetic results and adequate family counselling. Therefore, the evaluation of a SUD case should be performed by a multidisciplinary team of cardiologists, geneticists, pathologists, and probably psychosocial counsellors [1, 12, 15, 33]. With this combined expertise, it will be possible to obtain clinical and genetic data of the family in order to establish a diagnosis from a synopsis of all assessments. The variant’s segregation within the family, in addition to the medical history of the deceased, is the most important information for determination of its role in SUD cases.

Family studies are not only necessary for disease-variant segregation and to determine the cause of death, but also to identify relatives possessing an increased risk for cardiac disease or even sudden death [33, 34].

The appropriate interpretation of genetic variants is an important issue, because the underestimation of the variant’s pathogenicity could result in a possible cardiac event or even another sudden death in the victim’s family. In contrast, an overestimation of pathogenicity could cause unnecessary fear in allegedly affected families or, even worse, invasive interventions or needless lifestyle adjustments (e.g., no sports) and thus severely interfere with an individual’s quality of life. Therefore, evaluating the sequencing data using a threshold for allele frequency is one of the most important criteria in an accurate data analysis. For instance, 0.2%, which is based on the prevalence of hypertrophic cardiomyopathy (1:500) and 0.005% (1 in 20,000 alleles or 1 in 10,000 individuals), reflecting rare variants are two rational allele frequency filters. Filtering with such a low allele frequency is useful for assessing variants, but one has to keep in mind that potentially important variants and information with regard to genetic modifiers may be missed. These could play a role in a SCD case, but are too common to be detected with the filter applied.

For this reason, a multidisciplinary approach with a cardiological assessment and genetic investigation of possibly affected family members in combination with a post-mortem genetic screening has proven to be the best strategy for a proper management of these tragic cases.

## Conclusions

Genetic testing of 56 unrelated SUD cases revealed a total 53 rare protein-altering variants (MAF < 0.2%) classified as VUS or worse. Seven cases (12%) exhibited a clinically actionable variant (pathogenic or likely pathogenic, VUS potentially pathogenic) that would warrant cascade genetic screening in relatives.

Nevertheless, the large amount of VUS detected in our study clearly demonstrates the requirement for the intervention of an experienced multidisciplinary team as well as the development of specific forensic guidelines to enable appropriate interpretation of rare genetic variants.

## Limitation

The non-uniformity of autopsy and cardiopathology datasets and lack of comprehensive clinical evaluation of the deceased persons’ relatives resulted from the retrospective character of this study. Therefore, the combined yield of post-mortem genetic screening in addition to clinical investigation of the deceased person’s family remains unknown.

## Supplementary Information

Below is the link to the electronic supplementary material.

Supplementary file1 (XLSX 12.4 kb)

## References

[1] Stiles MK, Wilde AA, Abrams DJ (2020). 2020 APHRS/HRS expert consensus statement on the investigation of decedents with sudden unexplained death and patients with sudden cardiac arrest, and of their families. Heart Rhythm.

[2] de Vreede-Swagemakers JJ, Gorgels AP, Dubois-Arbouw WI (1997). Out-of-hospital cardiac arrest in the 1990’s: a population-based study in the Maastricht area on incidence, characteristics and survival. J Am Coll Cardiol.

[3] Chugh SS, Jui J, Gunson K (2004). Current burden of sudden cardiac death: multiple source surveillance versus retrospective death certificate-based review in a large U.S. community. J Am Coll Cardiol.

[4] Stecker EC, Reinier K, Marijon E (2014). Public health burden of sudden cardiac death in the United States. Circ Arrhythm Electrophysiol.

[5] Kong MH, Fonarow GC, Peterson ED (2011). Systematic review of the incidence of sudden cardiac death in the United States. J Am Coll Cardiol.

[6] Kitamura T, Iwami T, Kawamura T (2012). Nationwide improvements in survival from out-of-hospital cardiac arrest in Japan. Circulation.

[7] Winkel BG, Holst AG, Theilade J (2011). Nationwide study of sudden cardiac death in persons aged 1–35 years. Eur Heart J.

[8] Bagnall RD, Weintraub RG, Ingles J (2016). A Prospective Study of Sudden Cardiac Death among Children and Young Adults. N Engl J Med.

[9] Ackerman MJ, Priori SG, Willems S (2011). HRS/EHRA expert consensus statement on the state of genetic testing for the channelopathies and cardiomyopathies this document was developed as a partnership between the Heart Rhythm Society (HRS) and the European Heart Rhythm Association (EHRA). Heart Rhythm.

[10] Cerrone M, Priori SG (2011). Genetics of sudden death: focus on inherited channelopathies. Eur Heart J.

[11] Lahrouchi N, Raju H, Lodder EM (2017). Utility of post-mortem genetic testing in cases of sudden arrhythmic death syndrome. J Am Coll Cardiol.

[12] Fellmann F, van El CG, Charron P (2019). European recommendations integrating genetic testing into multidisciplinary management of sudden cardiac death. Eur J Hum Genet.

[13] Ackerman JP, Bartos DC, Kapplinger JD (2016). The promise and peril of precision medicine: phenotyping still matters most. Mayo Clin Proc.

[14] Ackerman MJ, Priori SG, Willems S (2011). HRS/EHRA expert consensus statement on the state of genetic testing for the channelopathies and cardiomyopathies: this document was developed as a partnership between the Heart Rhythm Society (HRS) and the European Heart Rhythm Association (EHRA). Europace.

[15] Semsarian C, Ingles J, Wilde AAM (2015). Sudden cardiac death in the young: the molecular autopsy and a practical approach to surviving relatives. Eur Heart J.

[16] Richards S, Aziz N, Bale S (2015). Standards and guidelines for the interpretation of sequence variants: a joint consensus recommendation of the American College of Medical Genetics and Genomics and the Association for Molecular Pathology. Genet Med.

[17] Ackerman MJ (2015). Genetic purgatory and the cardiac channelopathies: exposing the variants of uncertain/unknown significance issue. Heart Rhythm.

[18] Scheiper-Welling S, Körber S, Geisen C (2021). Genetic analysis of sudden unexpected death cases: evaluation of library preparation methods to handle heterogeneous sample material. Forensic Sci Int.

[19] Köffer J, Scheiper-Welling S, Verhoff MA (2021). Post-mortem genetic investigation of cardiac disease-associated genes in sudden infant death syndrome (SIDS) cases. Int J Legal Med.

[20] Karczewski KJ, Francioli LC, Tiao G (2020). The mutational constraint spectrum quantified from variation in 141,456 humans. Nature.

[21] Auton A, Brooks LD, Durbin RM (2015). A global reference for human genetic variation. Nature.

[22] Adzhubei IA, Schmidt S, Peshkin L (2010). A method and server for predicting damaging missense mutations. Nat Methods.

[23] Steinhaus R, Proft S, Schuelke M (2021). MutationTaster2021. Nucleic Acids Res.

[24] Kumar P, Henikoff S, Ng PC (2009). Predicting the effects of coding non-synonymous variants on protein function using the SIFT algorithm. Nat Protoc.

[25] Kircher M, Witten DM, Jain P (2014). A general framework for estimating the relative pathogenicity of human genetic variants. Nat Genet.

[26] Mellor G, Raju H, de Noronha SV (2014). Clinical characteristics and circumstances of death in the sudden arrhythmic death syndrome. Circ Arrhythm Electrophysiol.

[27] Michaud K, Grabherr S, Jackowski C (2014). Postmortem imaging of sudden cardiac death. Int J Legal Med.

[28] Coll M, Pérez-Serra A, Mates J et al. (2017) Incomplete penetrance and variable expressivity: hallmarks in channelopathies associated with sudden cardiac death. Biology (Basel) 7.10.3390/biology7010003 10.3390/biology7010003 PMC5872029 29278359

[29] Bezzina CR, Lahrouchi N, Priori SG (2015). Genetics of sudden cardiac death. Circ Res.

[30] Scheiper-Welling S, Zuccolini P, Rauh O (2020). Characterization of an N-terminal Nav1.5 channel variant - a potential risk factor for arrhythmias and sudden death?. BMC Med Genet.

[31] Jenewein T, Beckmann BM, Rose S (2017). Genotype-phenotype dilemma in a case of sudden cardiac death with the E1053K mutation and a deletion in the SCN5A gene. Forensic Sci Int.

[32] Jenewein T, Neumann T, Erkapic D (2018). Influence of genetic modifiers on sudden cardiac death cases. Int J Legal Med.

[33] Kauferstein S, Herz N, Scheiper S (2017). Relevance of molecular testing in patients with a family history of sudden death. Forensic Sci Int.

[34] Gray B, Ackerman MJ, Semsarian C (2019). Evaluation after sudden death in the young: a global approach. Circ Arrhythm Electrophysiol.

